# The Functional Difference of Dendritic Cells in HBeAg Negative Chronic Hepatitis B Patients with Three Different Spleen Deficiency Syndromes and the Therapeutic Evaluation of Chinese Medicine Intervention Based on Syndrome Differentiation 

**DOI:** 10.1155/2014/802402

**Published:** 2014-06-29

**Authors:** Lei Wang, Li Zhang, Xiaoxia Feng, Lianjun Xing, Wei Zhang, Kaiping Jiang, Haiyan Song, Guang Ji

**Affiliations:** ^1^Institute of Digestive Diseases, Longhua Hospital, Shanghai University of Traditional Chinese Medicine, Shanghai 200032, China; ^2^Department of Hepatology, Longhua Hospital, Shanghai University of Traditional Chinese Medicine, Shanghai 200032, China; ^3^Department of Hepatology, Foshan Hospital of Traditional Chinese Medicine, Guangdong 528000, China; ^4^E-Institute of Shanghai Municipal Education Commission, Shanghai University of Traditional Chinese Medicine, Shanghai 201203, China

## Abstract

*Objective*. To investigate the dendritic cells (DCs) maturity differences of HBeAg negative chronic hepatitis B (CHB) patients with different spleen deficiency (SD) syndromes and explore the role of syndrome differentiation in the therapeutic evaluation of Chinese medicine.* Methods*. 120 participants were recruited including three treatment groups in different SD syndrome categories as spleen deficiency with liver depression (SDLD), spleen deficiency with damp heat (SDDH), and spleen deficiency with kidney deficiency (SDKD) and one healthy control group; each group had 30 participants. Corresponding drugs were applied. The outcome measures included DC phenotype, liver function, IL-10, IL-12, and HBV-DNA levels.* Results.* The surface markers of mature DCs and cytokines levels were different in each group; the positive rate of CD80, CD1a, HLA-DR, and CD1a was the lowest in SDKD group. After 3-month intervention, the expression of CD80, CD86, CD1a, HLA-DR, and IL-12 significantly increased, while ALT, AST, and IL-10 significantly decreased (*P* < 0.05) in treatment groups. HBV-DNA level also significantly reduced in both SDKD and SDLD groups (*P* < 0.05).* Conclusions.* HBeAg negative patients had DCs dysmaturity, and there were differences between different SD syndromes. Chinese medicine intervention according to syndrome differentiation could advance the maturity and function of DCs and improve the therapeutic effect.

## 1. Introduction

Chronic hepatitis B (CHB) is a common infectious disease with high prevalence in the world, and about one million people die from hepatitis B virus (HBV) related hepatic failure, liver cirrhosis, and primary carcinoma annually [1, 2]. Other than HBV infection, immune deficiency of the host is also the vital reason for the continuous existence of hepatitis B virus [3, 4]. Numerous studies have shown that there are various degrees of immune tolerance in CHB patients. Dendritic cells (DCs) are the most powerful antigen presenting cells (APC) in human, and their maturity and function have direct influence on cellular and humoral immunity [5–7]. Besides the immune characteristic of CHB, HBeAg negative CHB also has the characteristics of low rate of antiviral treatment response, poor long-term prognosis, and high occurrence of hepatocellular carcinoma (HCC), which make the effects of conventional medical treatment unsatisfactory and make people seek for traditional Chinese medicine (TCM) to manage their symptoms [8].

According to TCM theory, syndrome differentiation is the critical step of disease diagnosis and treatment. In TCM, the spectrum of syndrome includes clinical information such as symptoms, signs, coating on the tongue, pulse condition, and the process to perceive this information. The prescriptions of the same disease may be different due to different syndromes. Studies have shown that the same disease with different TCM syndromes may have different substrates, and these differences might be helpful for exploring the Chinese syndrome classification and guiding the clinical treatment and therapeutic evaluation [9, 10]. Spleen deficiency (SD) is the most common syndrome for CHB patients. However, the syndromes of CHB patients are not always the same, and most patients' clinical presentation may combine with other syndromes such as “liver depression,” “damp heat,” and “kidney deficiency.” Researches of relationship between various syndrome phenotypes and disease characteristics will not only contribute to explain the scientific nature of Chinese syndrome classification, but also guide the strategy setting of the treatment and thus improve the clinical effects of Chinese treatment. According to the previous studies, SD is closely associated with immune dysfunction [11] and as DCs have the immunological characteristics, we hypothesized that there may be some differences of DC phenotype and function among various SD categories in HBeAg negative CHB patients, and these differences may influence the clinical effects of Chinese medicine treatment.

This study aims to assess the DC phenotype and function in different SD categories as well as other immune indexes (IL-10 and IL-12) among the HBeAg negative CHB patients based on the main therapy strategy of “treatment according to the syndrome.” We hope it will be helpful to explain the immunological characteristics of Chinese medicine syndrome and shed light on the scientific mechanism of syndrome differentiation to improve the effects of Chinese medicine interventions.

## 2. Materials and Methods

### 2.1. Study Design

This study applies a prospective cohort control study method, and, according to TCM criteria, HBeAg negative CHB patients with spleen deficiency are divided into SDLD group (group A), SDDH group (group B), and spleen SDKD group (group C) and the normal control group is regarded as group K.

### 2.2. Participants and Setting

The participants were screened from the outpatient clinic in the Department of Hepatology in Longhua Hospital. 30 healthy volunteers from Longhua Hospital attached to Shanghai University of TCM were selected as normal control group.

The inclusion criteria of treatment group included the following: (a) Chinese male or female aged from 18 to 60 years old, (b) having the diagnostic criteria of HBeAg negative CHB, according to Chinese Medicine Association “Chronic Hepatitis B Control Guideline, 2011 version,” and (c) meeting the syndrome differentiation standards of spleen deficiency and related syndromes (e.g., liver stagnation, spleen deficiency and dampness heat, and spleen deficiency and kidney deficiency) as listed below in detail. The exclusion criteria of treatment group included having not taken antiretroviral therapy and immune therapy within six months and having not received any other treatment within one month.

#### 2.2.1. Criteria of TCM Syndrome

The guiding principles of clinical study of new TCM medicine and the TCM syndrome classification standards in the fifth edition of TCM diagnostics were applied as our criteria of TCM of SD syndrome [12, 13]. Anyone who has two of the following symptoms: anorexia, fatigue, abdominal distention after eating, and abnormal stool, is diagnosed as having spleen deficiency syndrome. The subtypes of SD syndromes were listed as follows. (a) SDLD meets the diagnosis of spleen deficiency syndrome and has any three of the following symptoms: chest and hypochondrium; abdominal distention and pain; belching; acid regurgitation; depression or irritability; frequent sighing; dry and bitter mouth or obstruction in throat; and so on. (b) SDDH meets the diagnosis of spleen deficiency syndrome and has any three symptoms of thirst but no desire of water; loose stools; heavy limbs; dull fever; red tongue and slimy yellow fur; pulse and moist; and so on, so it is diagnosed. (c) SDKD meets the diagnosis of spleen deficiency syndrome and has any two symptoms of soreness and weakness of waist and knees; sexual hypoactivity; extreme chilliness; frequent urination at night; dysphoria in chestpalms-soles, so it is diagnosed. Every syndrome is recorded as 0, 1, 2, and 3, according to the severity. Two senior TCM professional physicians evaluated the syndrome independently, and only the consistent results were included.

The study was approved by the Institute Review Board of Shanghai University of TCM. All participants were given a written informed consent form and were free to withdraw from the trial at any time.

### 2.3. Interventions

Patients in group A were given ChaihuShugan in combination with Sijunzi decoction (*Codonopsis pilosula* 10 g,* Atractylodes macrocephala* Koidz. 9 g,* Poria cocos* 9 g,* Glycyrrhiza uralensis* 6 g,* Bupleurum chinense* 6 g,* Pericarpium citri* Reticulatae 6 g, Rhizoma Chuanxiong 4.5 g, Rhizoma Cyperi 4.5 g, Fructus Aurantii 4.5 g, and* Paeonia lactiflora* 4.5 g); patients in group B were given Yinchenhao decoction in combination with Sijunzi decoction (*Codonopsis pilosula* 10 g,* Atractylodes macrocephala* Koidz. 9 g,* Poria cocos* 9 g,* Glycyrrhiza uralensis* 6 g,* Artemisia capillaris* Thunb. 30 g,* Gardenia jasminoides* 15 g, and* Rheum palmatum* 9 g); patients in group C are given Liuwei Dihuang Wan or Jingui Dihuang Wanin combination with Sijunzi decoction (*Codonopsis pilosula* 10 g,* Atractylodes macrocephala* Koidz. 9 g,* Poria cocos* 9 g,* Glycyrrhiza uralensis* 6 g,* Rehmannia glutinosa* 24 g, fructus corni 12 g,* Dioscorea opposita* 12 g, Rhizoma Alismatis 9 g, Cortex Moutan 9 g,* Ramulus Cinnamomi* 9 g, and Aconitum carmichaeli Debx 3 g) (Table 1). The decoction was prepared by manufacturing laboratory in Longhua Hospital attached to Shanghai University of TCM in vacuum bags (150 mL/bag) and the patients were instructed to take the medicine (150 mL) twice a day for each prescription, and the duration of one-course treatment was three months.

### 2.4. Outcomes

#### 2.4.1. TCM Syndrome Score Calculation

The scores are recorded and marked as obviously effective: symptoms and signs are improved markedly, and symptom scores decrease ≥70%; effective: symptoms and signs are improved gradually, and symptom scores decrease ≥30%; invalid: symptoms and signs are not significantly improved, and symptom scores decrease ≤30%. The formula (nimodipine method) is as follows: [(total score before treatment − total score after treatment)/total score before treatment] × 100% [14].

#### 2.4.2. Reagents and Instruments

RPMI-1640 culture medium and fetal bovine serum were purchased from GIBCO (USA); granulocyte-macrophage colony stimulating factor (GM-CSF), recombinant human interleukin-4 (IL-4), and tumor necrosis factor-*α* (TNF-*α*) were obtained from Peprotech (USA); human interleukin-10 (IL-10), human interleukin-12 (IL-12), and ELISA kit were from R&D company (USA); fluorescence labeling CD80-FITC, CD86-PE, CD1a-PECY5, and HLA-DR-APC monoclonal antibodies were from BD Company (USA); FACSCalibur flow cytometry was also from BD Company (USA).

#### 2.4.3. The Liver Function and HBV-DNA Detection

Liver function detection applied biochemical rate method and was done by clinical laboratory in Longhua Hospital attached to Shanghai University of TCM. HBV-DNA was detected by fluorescent quantization and implemented by Shanghai Aidicang Medical Diagnostic Centre.

#### 2.4.4. Separation of Peripheral Blood Mononuclear Cell (PBMC)

The method was applied according to the literature [15, 16]. In brief, 10 mL peripheral blood is collected from each subject, under sterile conditions; after the heparin anticoagulation, same volume of PBS is added for dilution. Spread the mixture on the lymphocyte separation medium, centrifuge 800 g/min for 20 min at room temperature, collect the cells in the interface between upper and middle layer, add PBS for suspension and washing, add serum-free RPMI1640 medium to precipitate the cells, count and adjust the cell concentration to 2~6 × 10^6^/mL, add 1.5 mL for each group, put the cells into plates, incubate the cells for 2 hours in an incubator at 37°C with a humidified 5% CO_2_ atmosphere, remove nonadherent cells, and then get the peripheral blood mononuclear cells.

#### 2.4.5. Differentiation and Culture for DCs In Vitro

The method was applied according to the literature [17–19]. Wash mononuclear cells with serum-free RPMI 1640 for three times, add 10% fetal bovine serum RPMI 1640 medium, and add cytokine into the final concentration of 100 ng/mL and rhIL-4 100 ng/mL of rhGM-CSF. Continue to cultivate the cells and change the medium every 48 h (including the cytokine, rhGM-CSF 50 ng/mL, rhIL-4 50 ng/mL), and then add TNF-*α* (100 ng/mL) on the fifth day, incubate 48 hours, and collect DCs.

#### 2.4.6. DCs Morphology and Proliferation Observation

Observe DCs morphology and proliferation under phase-contrast microscope on the first, third, fifth, and seventh day and observe the morphology of mature DCs with scanning electron microscope.

#### 2.4.7. Detection of DCs Surface Markers

Cultivate the DCs for seven days and detect the surface molecules of DCs, such as CD80, CD86, and CD1a, and the expression level of HLA-DR with flow cytometry. All the procedures were conducted according to the kits [18].

#### 2.4.8. Detection of IL-10 and IL-12

Collect the DCs cell supernatant and detect the content of cytokines IL-10 and IL-12 with ELISA method.

### 2.5. Statistical Methods

Measurement data is represented by mean ± standard deviation (SD). HBV-DNA was compared after logarithmic transformation. Use variance analysis method and least significant difference (LSD) to compare the difference between groups and use paired *t*-test to compare the mean of each group before and after the treatment. All the data were analyzed by SPSS 18.0 software and a *P* value less than 0.05 was considered statistically significant.

## 3. Results

### 3.1. Baseline Data

**Figure 1:**
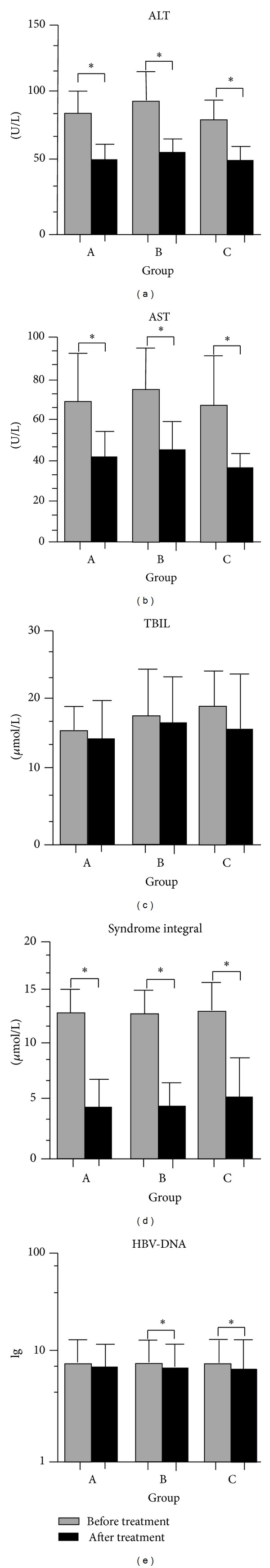
The effectiveness of treatment according to syndrome differentiation among HBeAg negative CHB patients. (a) Comparison of ALT levels before and after treatment in each group. (b) Comparison of AST levels before and after treatment in each group. (c) Comparison of TBIL levels before and after treatment in each group. (d) Comparison of syndrome scores before and after treatment in each group. (e) Comparison of HBV-DNA load before and after treatment in each group. 30 patients in each group; ^∗^indicates *P* < 0.05 between groups.

114 patients diagnosed with “spleen deficiency” were included, and 24 participants were excluded after syndrome screening. 90 HBeAg negative CHB patients were recruited from the outpatient clinic in the Department of Hepatology in Longhua Hospital from January 2012 to December 2012: 51 males (age range from 19 to 60 years; average age is 39.5 years), with 2–21 years disease course (average course is 9.8 years), and 39 females (age range from 20 to 60 years; average age is 44.3 years), with 2–20 years disease course (average course is 10.6 years). The comparative difference between gender, course of disease, liver function, and HBV-DNA levels of HBeAg negative CHB patients in three different spleen deficiency syndrome groups before treatment has no statistical significance (*P* > 0.05). The age of patients in group C was higher than that of group A and group B (*P* < 0.05) (Tables 2 and 3, Figure 1).

### 3.2. The Effects of Chinese Medicine Treatment according to Syndrome Differentiation among HBeAg Negative CHB

Compared with baseline, all the enzymes, reflecting liver function and ALT and AST levels in each group, decreased significantly (*P* < 0.05) (Table 3, Figures 1(a) and 1(b)) while the total bilirubin (TBIL) among groups had no significant difference (Table 3, Figure 1(c)). Similarly, HBV-DNA in group B and group C reduced significantly compared to that before treatment (*P* < 0.05) (Table 3, Figure 1(e)). The total syndrome scores in each group also improved significantly after treatment (*P* < 0.05). In group A, there were 10 obviously effective cases, 9 effective cases, and 1 invalid case. In group B, there were 7 obviously effective cases, 12 effective cases, and 1 invalid case. In group C, there were 8 obviously effective cases, 12 effective cases, and there was no significant difference regarding the therapeutic effect among groups (Table 3, Figure 1(d)).

### 3.3. DCs Morphology and Proliferation

**Figure 2:**
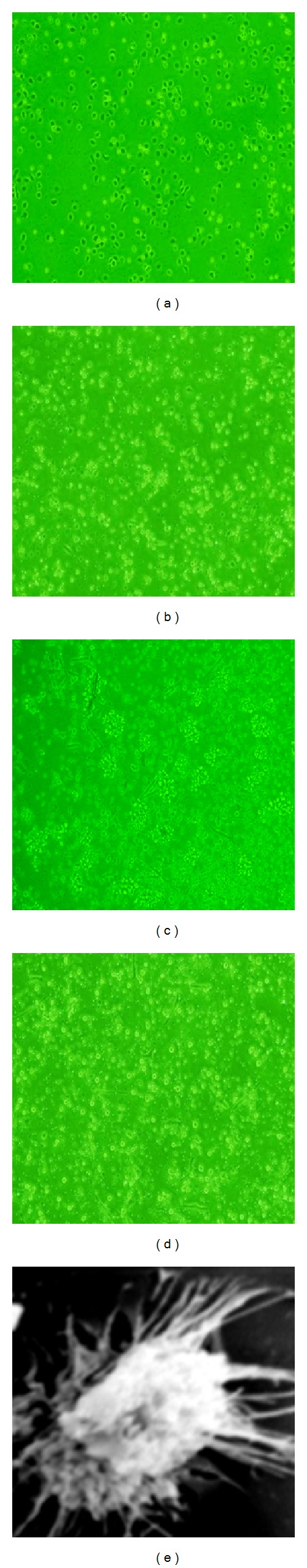
Morphological changes of DCs during different periods. (a) The second hour of culture. (b) The third day of culture. (c) On the fifth day of culture. (d) On the seventh day of culture. (e) On the seventh day, the DCs morphology was observed by scanning electron microscope.

The isolated PBMC displayed as quasicircular adherent mononuclear cells after 2 h still standing (Figure 2(a)). After 3-day coculture with cytokines, the suspended cells increased and adherent mononuclear cells grew aggregately (Figure 2(b)). Until the fifth day, the size of the cells increased and shaped into a light irregular appearance and the colony presented (Figure 2(c)). On the seventh day, the cells were suspended but could not attach the container; their shape was irregular and numerous extended burrs could be seen. From the further observation by scanning electron microscope, it represented the shape of mature DCs (Figures 2(d) and 2(e)). The DCs proliferation from CHB patients was relatively slower than that from healthy people.

### 3.4. The DC Phenotype and the Comparison of IL-10 and IL-12 of HBeAg Negative CHB Patients before and after Treatment

**Figure 3:**
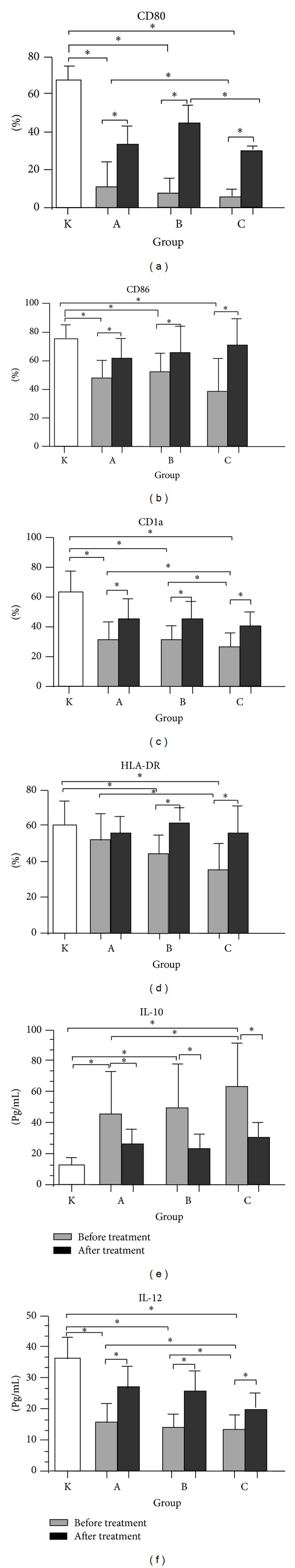
The change of DC phenotype and function of CHB patients in responding to treatment. (a) The comparison of CD80 before and after treatment in each group. (b) The comparison of CD86 before and after treatment in each group. (c) The comparison of CD1a before and after treatment in each group. (d) The comparison of HLA-DR before and after treatment in each group. (e) The comparison of IL-10 before and after treatment in each group. (f) The comparison of IL-12 before and after treatment in each group. 30 patient cases in each group; ^∗^indicates *P* < 0.05 between the corresponding groups.

Before treatment, the expression rate of DCs surface biomarkers of HBeAg negative CHB patients was significantly lower than healthy people (*P* < 0.05) and the expression level of DCs surface biomarkers between various syndrome types was different (Table 4). Specifically, the positive expression rate of CD80, CD1a, and HLA-DR in group A patients was higher than that of group C (*P* < 0.05), and the positive expression rate of CD1a in group B patients was higher than that of group C (*P* < 0.05). Moreover, the content of IL-10 in DCs supernatant of CHB patients was higher than healthy people, while the IL-12 content is lower (*P* < 0.05). IL-10 content in group C is significantly higher than that of group A, while IL-12 content is lower than that of group A and group B (*P* < 0.05). Compared to the baseline, the expression of CD80 (Table 4, Figure 3(a)), CD86 (Table 4, Figure 3(b)), CD1a (Table 4, Figure 3(c)), HLA-DR (Table 4, Figure 3(d)), and IL-12 (Table 4, Figure 3(f)) content after the treatment in each group significantly increased (*P* < 0.05), while IL-10 content decreased (Table 4, Figure 3(e)). After the treatment, the comparison of other surface markers of DCs has no statistical significance (Table 4, Figure 3), except that the expression of CD80 in group B is higher than that of group C.

## 4. Discussion

The HBV infection is a public health problem attracting global attention. The continuous infection mechanism of HBV is not completely defined, and the defect of host immune is regarded as an important mechanism [20]. DCs are the most potent antigen presenting cells, and studies showed that chronic HBV infected patients have the defect of DCs maturity and function, so it cannot deliver the signal of virus antigens to the immune system, which is one of the major mechanisms of chronicity of hepatitis B [21, 22]. Generally the differentiation and development of DCs go through two stages. Immature DCs have the ability of capturing and processing antigens, after taking in the antigens; they can spontaneously mature and obtain the ability of activating primary type T cells and complete immune stimulating function [23]. Compared with immature DCs, the antigen capture ability of mature DCs decreases, but their antigen presenting ability is obviously enhanced. Previous research has shown that the decreased number, immature phenotype, and defected function of DCs of peripheral blood in CHB patients caused the failure of antigens presenting to CD4+ adjuvanticity T lymphocytes (Th) and CD8+ cytotoxic T lymphocytes (CTLs) [24, 25].

Traditional Chinese medicine plays an indispensable role in CHB and even HCC treatment [26]. Not only is it an effective treatment method for patients who do not need antiviral therapy but also it can improve the immune function and have antiviral effect according to some research findings [27]. Based on long-term clinical practice and pilot observational study, our research team found that HBeAg negative CHB patients had a certain amount of “spleen deficiency” syndrome and also the syndrome of “liver depression,” “damp heat,” “kidney deficiency,” and so on. This different syndrome classification based on “spleen deficiency” had differences of basic immune level and medical treatment sensitivity. This clinical research, through observing the changes of patients' syndrome scores before and after treatment, evaluating the effectiveness of “syndrome differentiation” objectively, and illustrating the scientific mechanisms of different Chinese syndromes based on the research hypothesis of DC functional state, provided referable evidence for TCM in treating HBeAg negative CHB patients and helped the investigators to determine the appropriate therapies for HBeAg negative CHB patients based on the different Chinese medicine syndromes according to evidence-based medical treatment [28].

Our results also showed that there was no significant difference of gender distribution, course of disease, liver function, and HBV-DNA load before treatment among three different “spleen deficiency” syndromes of CHB patients, while the age of SDKD patients was older than that of SDLD and SDDH patients. This may be explained by TCM theory, where the appearance of “kidney deficiency” is associated with the increase of age and the course of disease is related to the progress of pathology. In our study, we observed that the age in SDKD group was older than that in the other two groups, which was consistent with the TCM theory. Meanwhile, the course of disease among the three groups had no statistic difference; thus, we considered that the diversity of age in our study was not the main factor to explain the difference of DC function and phenotype.

Our data also showed that TCM treatment according to syndrome differentiation could reduce both syndrome scores and ALT, AST levels compared to the baseline, demonstrating preferable therapeutic effect. Unexpectedly, we found that the HBV-DNA load in SDDH group and SDKD group also decreased after TCM treatment. In suppressing HBV-DNA replication, it is usually considered that nucleoside analogues and interferon are superior, while our data backed that proper TCM treatment (according to syndrome differentiation) might be an optional method to improve therapeutic effect in clinical practice.

**Figure 4:**
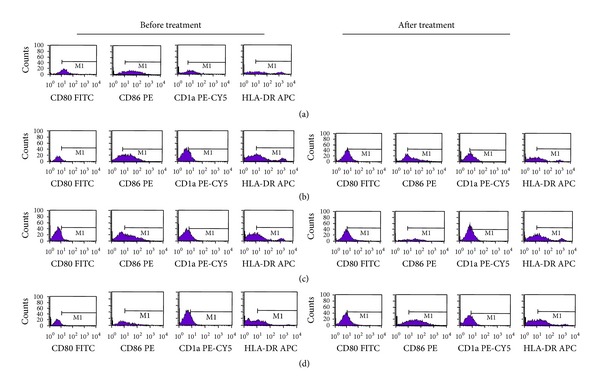
The change of DCs surface biomarkers before and after treatment in each group: Panel (a) CD80, CD86, CD1a, and HLR-DR expression in DCs from group K; Panel (b) CD80, CD86, CD1a, and HLR-DR expression in DCs from group A; Panel (c) CD80, CD86, CD1a, and HLR-DR expression in DCs from group B; Panel (d) CD80, CD86, CD1a, and HLR-DR expression in DCs from group C.

The change of DC function and phenotype is one of the important immune mechanisms in HBeAg negative CHB [21]. Our study observed the change of DCs surface biomarkers including CD80, CD86, CD1a, and HLA-DR and secreted cytokines IL-10, IL-12 (Figure 4). Before treatment, DC surface biomarkers expression was different among the three spleen deficiency syndromes in HBeAg negative CHB patients; the expression of CD80, CD1a, and HLA-DR in SDLD group and the expression of CD1a in SDDH group are higher than those in SDKD group, and the IL-10 content of DCs supernatant in SDKD group is higher than that in SDLD group, while IL-12 content is lower than that in SDLD and SDDH groups. IL-10 is an inhibitory cytokine, and it can decrease the antigen presenting function of DCs, through prohibiting the differentiation and maturity of DCs and inhibiting the secretion of IL-12 [29]. Our results demonstrated the differences of biomarkers expression among the three different spleen deficiency syndromes, indicating some scientific mechanism of the classification syndrome in HBeAg negative CHB patients. In our study, both SDLD and SDDH belong to the intermingling syndrome (deficiency and excess combination), so the biomarkers have no significant difference. The DC function and phenotype might be insufficient to distinguish the three different spleen deficiency syndromes, and other characteristic biomarkers are needed to further make the classification of syndromes. After syndrome targeting TCM treatment, the maturity and function of DCs were improved; the syndrome scores and HBV-DNA load all reduced.

Although our findings indicated that Chinese medicine intervention according to syndrome differentiation could advance the maturity and function of DCs as well as other immune indexes, the tentatively positive effects shown in the present study should be interpreted with caution. This study has a number of limitations. Firstly, this is a cohort study without any randomization, which made the self-selection bias obviously present, and the investigators and assessors also may have personal bias in the research outcome measurements. Secondly, this study's sample size is not enough and only 120 participants with 30 in each group which may not be able to represent the real situation of the syndrome distribution and effective size. Thirdly, the quality control of the herbal decoction could not be well controlled as the granules and we would use the high quality control herbal granules in next studies. Fourthly, our study was a small single-centre trial in a single ethnic Chinese population; thus, our findings may not be broadly generalizable.

## 5. Conclusion

Our research showed that TCM therapy based on syndrome differentiation could improve therapeutic effect of HBeAg negative CHB patients in DC phenotype, liver function, interleukin-10 (IL-10), IL-12, and HBV-DNA levels before and after the treatment. This result might provide some evidence in the rationale of syndrome-based treatments and further large-scale multicenter clinical studies on more diverse population are needed to confirm these findings.

## Figures and Tables

**Table 1:** Composition of the formula.

Group	SD category	Decoction name	Chinese herbs
A	SDLD	ChaihuShugan and Sijunzi decoction	*Codonops is pilosula *10 g, *Atractylodes macrocephala* Koidz. 9 g, *Poria cocos* 9 g, *Glycyrrhiza uralensis* 6 g, *Bupleurum chinense* 6 g, *Pericarpium citri* Reticulatae 6 g, Rhizoma Chuanxiong 4.5 g, Rhizoma Cyperi 4.5 g, Fructus Aurantii 4.5 g, *Paeonia lactiflora* 4.5 g

B	SDDH	Yinchenhao and Sijunzi decoction	*Codonops is pilosula* 10 g, *Atractylodes macrocephala* Koidz. 9 g, *Poria cocos* 9 g, *Glycyrrhiza uralensis* 6 g, *Artemisia capillaris* Thunb. 30 g, *Gardenia jasminoides* 15 g, *Rheum palmatum* 9 g

C	SDKD	Liuwei Dihuang Wan or Jingui Dihuang Wanin combination with Sijunzi decoction	*Codonopsispilosula* 10 g, *Atractylodes macrocephala* Koidz. 9 g, *Poriacocos* 9 g, *Glycyrrhiza uralensis* 6 g, *Rehmannia glutinosa* 24 g, *fructus corni *12 g, *Dioscorea opposita* 12 g, Rhizoma Alismatis 9 g, Cortex Moutan 9 g, Ramulus Cinnamomi 9 g, *Aconitum carmichaeli* Debx 3 g

**Table 2:** The comparison of gender, age, and course of disease of HBeAg negative CHB patients at baseline.

Group	Gender (*n*)		Age (year)	Course (year)
	Male	Female		
A	18	12	38.15 ± 6.18*	8.56 ± 3.45
B	17	13	37.65 ± 7.09*	9.43 ± 4.38
C	16	14	46.35 ± 10.34	11.73 ± 4.43

*P < 0.05, compared to group C.

**Table 3:** The comparison of liver enzymes and HBV-DNA levels in HBeAg negative CHB patients before and after treatment.

Group	ALT (U/L)	AST (U/L)	TBIL (umol/L)	HBV-DNA (IU/ML)
A				
Before	85.34 ± 15.14	71.26 ± 22.34	14.68 ± 4.34	2.23*E* + 7 ± 4.35*E* + 5
After	50.36 ± 8.53^#^	43.78 ± 11.25^#^	13.53 ± 6.29	1.98*E* + 7 ± 2.97*E* + 6
B				
Before	91.06 ± 16.20	77.13 ± 17.21	17.54 ± 6.85	1.47*E* + 7 ± 3.96*E* + 5
After	54.67 ± 9.15^#^	48.56 ± 13.21^#^	15.83 ± 7.61	4.32*E* + 6 ± 1.24*E* + 5*
C				
Before	83.94 ± 17.53	69.38 ± 23.16	18.65 ± 5.38	2.30*E* + 7 ± 4.63*E* + 5
After	49.86 ± 7.33^#^	39.66 ± 9.64^#^	14.79 ± 8.35	8.73*E* + 6 ± 4.16*E* + 5*

*P < 0.05, compared to group C; ^#^ *P* < 0.05, compared to baseline.

**Table 4:** The comparison of DCs biomarkers, IL-10, and IL-12 levels in HBeAg negative CHB patients before and after treatment.

Group	CD80 (%)	CD86 (%)	CD1a (%)	HLA-DR (%)	IL-10 (pg/mL)	IL-12 (pg/mL)
K	65.03 ± 9.25	75.60 ± 7.26	63.21 ± 15.43	59.21 ± 15.14	14.31 ± 6.22	36.67 ± 8.23
A						
Before	12.04 ± 10.54^#&^	49.51 ± 13.35^#^	32.37 ± 10.06^#&^	52.32 ± 16.01^&^	47.43 ± 24.59^#&^	16.25 ± 6.12^#&^
After	35.20 ± 10.47*	64.08 ± 11.21*	45.15 ± 11.31*	53.83 ± 12.99	24.88 ± 12.06*	26.12 ± 9.32*
B						
Before	9.13 ± 6.95^#^	51.38 ± 13.74^#^	30.11 ± 10.58^#&^	43.26 ± 16.35^#^	51.43 ± 24.41^#^	14.49 ± 5.03^#&^
After	42.66 ± 13.09^▲∗^	68.14 ± 15.97*	44.52 ± 10.63*	57.34 ± 10.68*	23.13 ± 11.40*	25.49 ± 8.42*
C						
Before	6.01 ± 3.32^#^	43.54 ± 20.06^#^	20.24 ± 9.37^#^	38.20 ± 14.12^#^	65.54 ± 27.18^#^	8.69 ± 4.13^#^
After	28.46 ± 7.90*	70.86 ± 18.42*	38.43 ± 12.19*	55.81 ± 13.88*	28.56 ± 9.78*	20.54 ± 7.47*

^#^ *P* < 0.01, compared to group K before treatment; ^&^ *P* < 0.05, compared to group C before treatment; *P < 0.05, compared to before treatment in each group; ^*▲*^ *P* < 0.05, compared to group C after treatment.
